# Application of Patient-Specific iPSCs for Modelling and Treatment of X-Linked Cardiomyopathies

**DOI:** 10.3390/ijms22158132

**Published:** 2021-07-29

**Authors:** Jennifer Zhang, Oscar Hou-In Chou, Yiu-Lam Tse, Kwong-Man Ng, Hung-Fat Tse

**Affiliations:** 1 Cardiology Division, Department of Medicine, Li Ka Shing Faculty of Medicine, The University of Hong Kong, Hong Kong, China; u3555558@connect.hku.hk (J.Z.); oscarjx1@connect.hku.hk (O.H.-I.C.); yltse2@hku.hk (Y.-L.T.); 2 Centre of Translational Stem Cell Biology, Hong Kong Science and Technology Park, Hong Kong, China

**Keywords:** X-linked cardiomyopathy, patient-specific induced pluripotent stem cells, disease modelling, drug screening

## Abstract

Inherited cardiomyopathies are among the major causes of heart failure and associated with significant mortality and morbidity. Currently, over 70 genes have been linked to the etiology of various forms of cardiomyopathy, some of which are X-linked. Due to the lack of appropriate cell and animal models, it has been difficult to model these X-linked cardiomyopathies. With the advancement of induced pluripotent stem cell (iPSC) technology, the ability to generate iPSC lines from patients with X-linked cardiomyopathy has facilitated in vitro modelling and drug testing for the condition. Nonetheless, due to the mosaicism of the X-chromosome inactivation, disease phenotypes of X-linked cardiomyopathy in heterozygous females are also usually more heterogeneous, with a broad spectrum of presentation. Recent advancements in iPSC procedures have enabled the isolation of cells with different lyonisation to generate isogenic disease and control cell lines. In this review, we will summarise the current strategies and examples of using an iPSC-based model to study different types of X-linked cardiomyopathy. The potential application of isogenic iPSC lines derived from a female patient with heterozygous Danon disease and drug screening will be demonstrated by our preliminary data. The limitations of an iPSC-derived cardiomyocyte-based platform will also be addressed.

## 1. Introduction

Cardiomyopathy is a highly heterogeneous myocardial disease that may contribute to the development of heart failure. In general, cardiomyopathies can be inherited or acquired. To date, more than 70 genes have been identified that are associated with various forms of cardiomyopathies, some of which (Table 1) are X-linked. Due to the substantial differences in the cardiac physiology of humans and rodents, the establishment of a disease model for X-linked cardiomyopathy has been difficult. For example, rodents have a rapid heart rate (300–800 bpm, compared with 60–100 bpm in humans) and their hearts have adapted to this with rapid systolic and diastolic filling [1]. Consequently, the action potentials of mice cardiomyocytes lack the plateau phase containing a sustained calcium release [2]. In addition, a recent study also showed that rodents lack the delayed rectifier potassium currents and show different responses to ion channel blockers compared with large animals and humans [3]. These cellular and electrophysiological differences may limit the usefulness of rodent models in studying X-linked cardiomyopathy [4]. With technical advancements in cellular reprogramming, adult somatic cells can now be reprogrammed into pluripotent stem cells [5,6,7], and such induced pluripotent stem cells (iPSCs) can be further differentiated into cardiomyocytes for disease modelling and drug testing [8]. To date, various studies have evaluated the possibility of using patient-specific iPSCs to model different forms of X-linked cardiomyopathy. In addition, there are some gender-specific differences in an iPSC model of X-linked cardiomyopathies. Apart from the different transmission pattern, the phenotypic expression in heterozygous females is usually more heterogeneous due to random X-chromosome inactivation. Due to the presence of a population of wildtype-like cells in the myocardium, heterozygous females with an X-linked dominant disorder (e.g., Danon disease) usually show a later disease onset and less severe symptoms than their male counterparts [9]. Disease phenotypes may also be observed in the heterozygous female carrying an X-linked recessive gene. Strategies, such as X-chromosome re-activation, have been demonstrated on iPSC platforms to solve these issues. In this review, the current strategies and examples of using patient-specific iPSC technology in modelling X-linked cardiomyopathies will be discussed. Our preliminary data using isogenic iPSC lines for drug screening will also be presented. Finally, the limitations of an iPSC modelling platform will be addressed.

## 2. The Advantages of an iPSC-Based Model

Most stem cells are of embryonic origin or derived from adult tissues with the ability to proliferate (e.g., bone marrow). These stem cells differentiate into a myriad of cell types but do not undergo a de-differentiation process [30]. Induced pluripotent stem cells can be reprogrammed directly from terminally differentiated somatic cells by over-expression of the four transcription factors (Oct4, Sox2, Klf4, and cMyc). The technology was first introduced in 2006 by Yamanaka et al. [7], and since then, iPSCs derived from patients or healthy individuals have been widely used for modelling various inherited disorders. Research in regenerative medicine is possible because of the ability of iPSCs to proliferate indefinitely and differentiate into various cell types, such as neurons, hepatocytes, and cardiomyocytes.

Since iPSCs are human in origin and pluripotent, they have enabled the development of patient-specific cell models to recapitulate the pathophysiology of human diseases [8]. This facilitates the study of inherited diseases, since the derived iPSCs and their differentiated cells will contain the same genomic profile as their patient somatic cell origin [31]. Additionally, novel drugs specific to the patient’s disease can be screened and tested on these disease-expressing cell types. For example, iPSC-derived cardiomyocytes can be used to reproduce the phenotype of autophagic dysfunction in cardiomyopathy caused by *LAMP2* mutation [32]; this also allows the identification of novel drugs that can reactivate the silenced X-chromosome.

An iPSC-based model has multiple advantages compared to the traditional cell lines. The traditional primary cell lines were difficult to establish from patients, especially for cell types that were difficult to access clinically. For instance, there are limited scenarios where human adult cardiomyocytes can be isolated, so most cultured cardiomyocytes have been isolated from neonatal cardiomyocytes [33]. Nonetheless neonatal cardiomyocytes express an electrophysiological and proteomics profile that differs from that of their adult counterparts. The iPSC-based model enables the derivation of a broad range of cell types from a readily accessible somatic cell source, such as blood cells and skin fibroblasts [34].

In the early years, a disease iPSC line would be paired with a control cell line derived from an individual without the disease. Nonetheless, with the advancement of gene-editing techniques, iPSC models can now be genetically manipulated to study the disease phenotype [35]. The development of the CRISPR-Cas9 system has enabled precise and specific gene-editing and epigenetic modulations [36,37]. Along with the expandable self-renewal features of iPSCs, the CRISPR-Cas9 system allows the induction of genetic changes in the targeted cell line, such as gene knockout/knock-in, in order to control gene expression [38]. In disease modelling, knockout of the disease-causing gene facilitates the establishment of an isogenic control cell line differing only in the disease-relevant gene [39]. Gene-editing can also introduce disease-causing mutations to a normal cell line to study the disease phenotype. Consequently, diverse genetically defective diseases can be modelled and help in the understanding of multiple genetic conditions [40].

The unique features of iPSCs provide many potential opportunities in disease modelling. An iPSC-based system allows for the development of a 3D platform to form self-organised organoids with intensive cell–cell interactions. These iPSC-derived organoids form primary tissue-like clumps that contain multiple cell types similar to their natural human counterpart [41]. This has advantages over the 2D culture platform, since the 3D organoids are expected to be more mature [42]. Apart from forming organoids, iPSCs also enable the formation of human–animal chimaera models via xenotransplantation. Engrafted cells can interact with the host microenvironment, replicating the diseased organ’s typical environment. Examples include the development of solid organ and neural chimaeras in order to study different diseases and model conditions, such as familial hypercholesterolemia and autistic spectrum disorders (ASD) [43,44].

## 3. The Procedures for Producing iPSCs

The fundamental principle of iPSC production is to express the reprogramming Yamanaka factors (Oct3/4, Sox2, c-Myc, and Klf4) in cells in order to drive the transcriptome into the pluripotent stage. Theoretically, iPSCs can be reprogrammed from any somatic cells with a complete genome. Although fibroblasts derived from skin biopsy have been widely used to generate iPSCs, collection is invasive and painful; thus, various alternatives are now available, including peripheral blood mononuclear cells (PBMC) from a blood sample and renal epithelial cells from urine [45,46,47,48].

The methods of delivering the factors into the somatic cells dictate the outcomes of the iPSCs. The first method involves the use of a retrovirus to transduce fibroblasts, but viral integration has been a huge issue [7]. Integration-free systems include either using the non-integrating virus, excisable transduction system, or the delivery of recombinant reprogramming proteins. Using lentiviral vectors instead of the retrovirus results in a higher efficiency of reprogramming with fewer viral integrations [49]. Such a simpler approach eliminates the risk of the possible integration of the virus genes into the host genome, so the resultant iPSC lines are more clinically relevant. Although integration-free systems can successfully reprogram somatic cells into iPSCs with equal quality, they are less efficient than the conventional integrating vectors, owing to the less sustainable reprogramming expression.

The iPSC was traditionally cultured on serum-dependent feeder layers comprised of either mouse or human fibroblasts [45]. Nonetheless the use of animal serum raised concerns about possible xeno-contamination [50]. Over time, iPSCs have been cultured in feeder-free conditions using components such as Matrigel, Geltrex, and other extracellular matrix proteins. Most iPSCs are now cultured in Essential 8™ or MTeSR™ medium; both systems maintain iPSC pluripotency after many passages and retain the ability to differentiate without causing any chromosomal aberrations [51].

**Figure 1:**
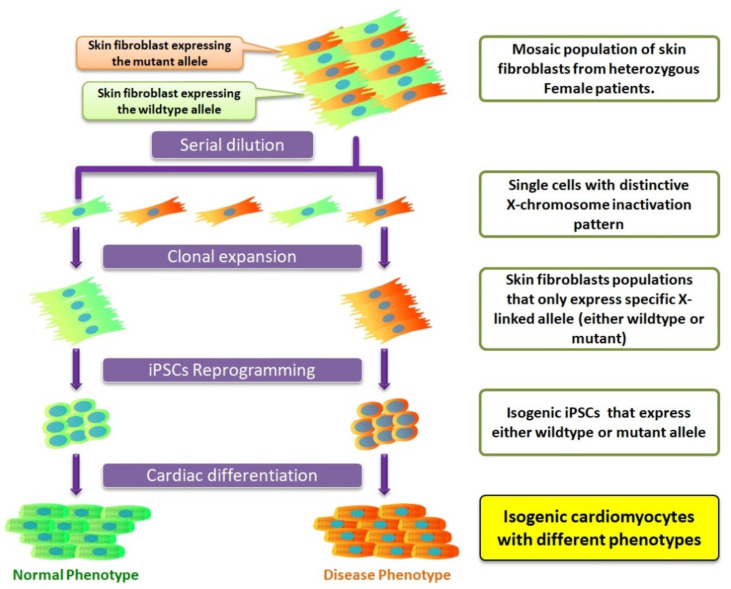
Generation of isogenic induced pluripotent stem cells (iPSCs) with distinctive X-chromosome inactivation status from the heterozygous female with X-linked mutation.

Refering to X-linked disorders, for male patients with only one X-chromosome in the genome, the iPSC line can usually be routinely generated and the cell lines produced will contain the mutation. Nevertheless, if the patient is a heterozygous female, due to the mosaic patten of X-chromosome inactivation, the generation of iPSCs with a specific active allele is not guaranteed. Based on our observation that the X-chromosome inactivation status remains unchanged during reprogramming, one should consider separating cells (skin fibroblasts or PBMCs) with a different X-chromosome inactivation status prior to transduction of the reprogramming factors. This can be achieved by diluting the cells to single cells and then checking the expression of the wildtype or mutant allele of interest using immunostaining or mRNA-based methods. (Figure 1). Using this approach, we created an isogenic pair of iPSCs that were genetically identical, differing only in their expression of the specific *LAMP2* allele, and used it to model Danon disease [7].

## 4. Differentiation of Established iPSC Lines to Cardiomyocytes

Differentiation protocols of established iPSC lines to cardiomyocytes have progressed from early differentiation of only 8–22% efficiency to current methods that achieve a high yield of cardiac troponin-T positive cells [52,53]. Earlier approaches included the use of embryoid bodies with serum-containing media, but their efficiency was very low [52]. Later efficient protocols included cardiac induction using cytokines with one of three established methods: suspended embryoid bodies, forced aggregation, or cell monolayer [54]. The first stage of cardiac differentiation mimics embryonic signals to induce mesodermal development [55,56,57,58,59], and the second step is cardiac specification [55,60,61,62]. For the maintenance of differentiated cardiomyocytes, most efficient protocols consist of basal medium RPMI 1640 supplemented with B27 serum or fetal bovine serum (FBS) at various concentrations [63,64,65]. Isolation of cardiomyocytes can be easily achieved using a glucose-depleted culture medium containing abundant lactate, since other cell types are not capable of using lactate for energy production. Another method of cardiomyocyte isolation includes cell sorting using antibodies against cardiomyocyte cell surface markers, such as SIRPa and VCAM1 [66,67,68,69].

**Figure 2:**
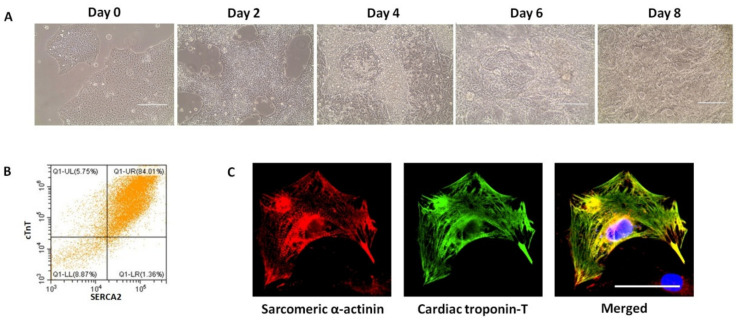
Differentiation of iPSCs into cardiomyocytes using commercially available kits from Thermo Fisher scientific: (**A**) the iPSCs were cultured as a monolayer in differentiation medium A (day 0-day 2), medium B (day 2-day 4), and cardiomyocyte maintenance medium (from day 4). Beating clusters usually can be observed by day 8, scale bar: 200 µm; (**B**) flow cytometry analysis indicates that the cardiac troponin-T (cTnT)-SERCA2-double positive cells constitute approximately 84%; (**C**) immunostaining of the cardiomyocytes dissociated from the beating clusters, scale bar: 50 µm.

The current differentiation protocol generates a heterogeneous group of cardiomyocytes. The addition of specific molecules has been shown to promote or inhibit certain cardiomyocyte subtypes in order to generate more specific disease models and drug testing platforms [70]. Studies show that the induction of appropriate mesoderm with different concentrations of small molecules to activate or inhibit certain signalling pathways specifically yields atrial, ventricular, or pacemaker-type cardiomyocytes [71,72,73,74,75,76,77,78]. Cardiomyocyte differentiation kits are now commercially available. Although their components may vary, they are convenient alternatives for laboratories that are routinely performing cardiac differentiation. For example, we are using the Gibco^TM^ PSC Cardiomyocyte Differentiation Kit from Thermo Fisher Scientific with a differentiation efficiency of 30–90%, depending on the genetic background of the iPSCs. For wildtype iPSCs, we consistently yield over 80% of cTNT positive cells (Figure 2).

## 5. Functional Characterisation of Cardiomyocytes Derived from iPSCs

Depending on the gene involved, the effect of a specific mutation on the cardiac phenotype may require individual evaluation. In general, the characterisation of patient-specific iPSC-derived cardiomyocytes should involve the structural organisation and electrophysiological and calcium handling properties, as well as contractile function.

### 5.1. Characterisation of Structural Properties

Cardiomyopathy patients usually exhibit characteristic changes in the heart tissue. For example, many X-linked mutations lead to hypertrophic phenotypes [13,17]. Nonetheless, it should be noted that iPSC-derived cardiomyocytes are usually irregular in shape. Although not many X-linked cardiomyopathy-associated genes are directly involved in cytoskeleton protein production, many mutations can precipitate disruption of the cytoskeleton organisation as a secondary effect [79]. As such, the examination of sarcomeric protein organisation may help to evaluate the pathophysiological effects of different mutations. Immunofluorescent microscopy can be used to identify pathological changes, such as disorganised myofilaments, by staining with antibodies specific to the sarcomeric proteins (e.g., sarcomeric α-actinin and cardiac-troponin-T) [32]. Electron microscopy with a resolution power of 50–200 pm can also identify phenotypic changes at an ultra-structural level [80]. Abnormalities in membranous structures, as well as glycogen granule accumulation, can be revealed, and are extremely useful in the evaluation of cardiac defects resulting from protein aggregation, autophagic dysfunction (Danon disease), and dysregulated intracellular storage (Danon disease and Fabry disease).

### 5.2. Characterisation of Electrophysiological Properties

Although no ion channel-encoding genes are located on the X-chromosome, mutations in X-linked genes, such as *MeCP2* [81], may indirectly affect cardiac electrophysiology; as such, it is essential to evaluate changes in electrophysiological properties in iPSC-derived cardiomyocytes when modelling X-linked cardiomyopathy. Patch clamp analysis has been used routinely to record the physiological properties of neurons and cardiomyocytes [82]. This method allows for the real-time measurement of changes in the membrane potential or electrical current of a single cell, a technique that requires great skill and is labour intensive and time consuming. Furthermore, since patch-clamp analysis works only on a single cell, it may not reveal the phenotypes related to conduction abnormality [83].

**Figure 3:**
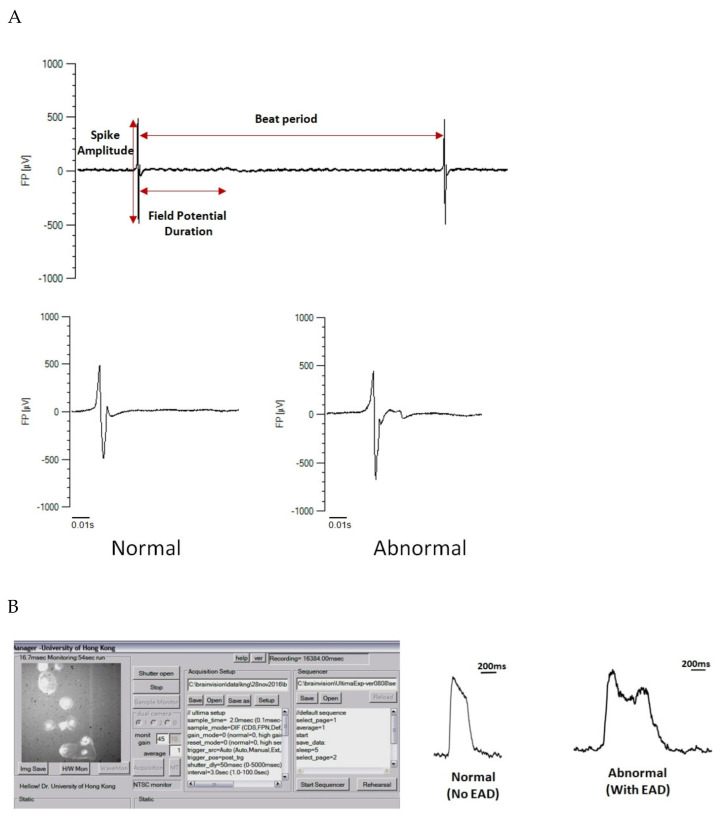
MEA and optical mapping for studying the electrophysiology of the iPSC-derived cardiomyocytes: (**A**) microelectrode array (MEA) analysis for evaluating the field potential changes across the monolayer surface of iPSC-derived cardiomyocytes; (**B**) optical mapping-based methods for evaluating the changes in membrane potentials of a single iPSC-derived cardiomyocyte. EAD: early after depolarisation.

Multielectrode arrays (MEA) analysis is another means of investigating the electrophysiology of cardiomyocytes. It enables the investigation of the mutations that induce conduction system defects [84]. By culturing a monolayer of iPSC-derived cardiomyocytes on the MEA plate, voltage changes due to depolarisation and repolarisation of cardiomyocytes across the surface can be simultaneously recorded to reveal the action potential conduction velocity [85]. As well as MEA, optical mapping (OM) can be used to study disease phenotypes associated with propagation abnormalities [86]. Although this method was developed to visualise myocardial infarction-induced heart block in isolated animal hearts, it can be modified to record the conduction velocity across the monolayers of iPSC-derived cardiomyocytes (Figure 3A). Unlike the MEA system, the OM system does not directly record the voltage changes with electrodes; instead, a voltage-sensitive fluorochrome (e.g., Di-8-ANEPPS) is used as the voltage indicator to provide a highly sensitive recording of voltage changes across the cardiomyocytes (Figure 3B).

### 5.3. Characterisation of the Calcium Handling Properties

During excitation–contraction coupling, the release and reuptake of sarcomeric calcium relays the electric signalling and the consequent contraction of cardiomyocytes. Failure in the regulation or handling of intracellular calcium may significantly compromise cardiac function or lead to cardiomyocyte death [87]. Again, mutations in X-linked genes, such as *LAMP2*, can indirectly alter intracellular calcium handling [32]; thus, the study of intracellular calcium handling is important to understand the pathophysiology of X-linked cardiomyopathies. Currently, the most widely used method to study intracellular calcium handling involves the use of calcium-sensing fluorochromes (e.g., Fura-2-AM) to trace the intracellular calcium transient. Upon electrical stimulation, the release of intracellular calcium is indicated by the fluorescent emission that is recorded with an ultra-fast and sensitive camera [81].

### 5.4. Characterisations of Contraction Properties

**Figure 4:**
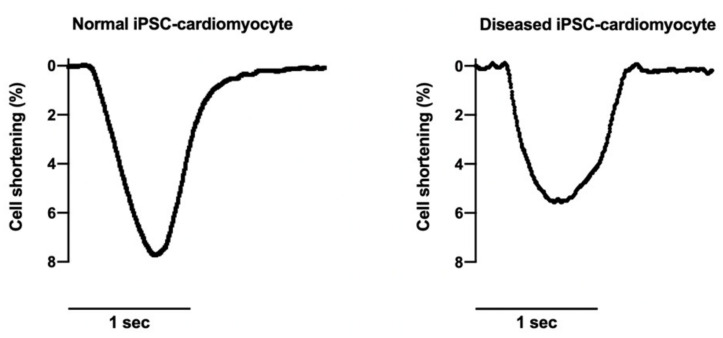
IonOptix recording of the change in cell length during contraction.

Measurement of the contractile property of an iPSC-derived cardiomyocyte is a direct indication of the effects of a mutation on cardiac function [88]. This is especially important when evaluating the effects of mutations in the genes encoding cytoskeleton networks (e.g., *FLNA*). Contractile function can be evaluated by different methods. The IonOptix cardiomyocyte contraction system allows for a high-speed recording (1000 hz) of the shortening and re-lengthening of a single cardiomyocyte (Figure 4). Nevertheless, since iPSC-derived cardiomyocytes are usually of irregular shape and contract multi-directionally, some laboratories may prefer to measure the contractile force generated during the contraction of the iPSC-derived cardiomyocyte with anatomic force microscopy [89].

## 6. Strategies for Using iPSC-Based Models for Disease Modelling and Drug Testing

The iPSC-derived cardiomyocyte-based model is a simple, reproducible, and economically efficient platform for disease modelling and drug testing [90]. The iPSC-derived cardiomyocytes demonstrate characteristic electrophysiological functions and pharmacological responsiveness comparable to primary cardiomyocytes, and thus have been used extensively for disease modelling and drug testing (Table 2 and Table 3) [61]. Patient-specific iPSCs have been used to reproduce a myriad of cardiomyopathy phenotypes, including long QT syndrome, Brugada syndrome, and familial dilated cardiomyopathy [91,92,93]. The electrophysiological indices of different ion channel blockers have been assessed for their antiarrhythmic properties and proarrhythmic risks using an aniPSC-derived cardiomyocyte-based platform using a multi-electrode array system [94,95,96]. Precision medicine, such as gender and population-specific differences in drug responses, can also be tested with iPSC-derived cardiomyocytes [97].

Sub-population-specific cardiomyocytes can be used for disease modelling and drug testing in pathology involving specific subtypes only [98]. Atrial-selective pharmacology is a potential means to treat atrial fibrillation with fewer side effects; drugs specific to atrial ion channels have been shown to reduce early repolarisation of atrial but not ventricular cardiomyocytes [99]. Atrial-specific cardiomyocytes have been used to assess atrial selectivity and efficacy of antiarrhythmic drugs [99,100]. Drug-induced arrhythmia has also been tested with ventricular-like cardiomyocytes [101].

Traditional two-dimensional cell cultures have limited translational capacity to humans. Consequently, the development of human cardiac organoid technologies may mimic the tissue microenvironment and increase confidence in translatability in drug screening and disease modelling [102]. Some 3D cultures show a more mature electrophysiological function, comparable to the normal adult myocardium [103,104,105]. A 3D model from a patient-derived iPSC for cardio-facio-cutaneous syndrome demonstrated hypertrophic cardiomyopathy-related disease phenotypes in cardiac tissues that could not be shown with 2D cultures [106]. Other conditions, such as Barth syndrome, inherited dilated cardiomyopathy, and arrhythmia, have also been modelled using 3D organoids [107,108,109]. An iPSC-derived 3D human engineered cardiac tissue model has also been used to study the potential of gene therapy in restoring the dystrophin expression and mechanical contraction force in DMD [110].

Furthermore, iPSC-derived cardiomyocyte-based cardiac repair is a promising treatment for the injured heart. Allogenic transplantation of iPSC-derived cardiomyocytes has been shown to improve cardiac contractile function and attenuate ventricular dilation in monkeys, mice, and rats with myocardial infarction, without inducing immune rejection [111,112,113]. Infarct size and apoptosis have both been reported to be reduced with the transplantation of patches of iPSC-derived cardiomyocytes that have also been shown to have additional cytoprotective and angiogenesis effects in order to improve the survival of damaged myocardium [114,115]. The improved cardiac function is likely due to the formation of mature grafts by injected iPSC-CMs that continuously express cardiac markers and sarcomere structures [116].

### 6.1. Duchenne Muscular Dystrophy

Duchenne muscular dystrophy (DMD) is an X-linked condition affecting 19.8 males in every 100,000 and resulting in progressive muscle wasting [132]. It is caused by a *DMD* gene mutation [133]. The *DMD* gene is located on the short arm of the X-chromosome and codes for the dystrophin protein. Frame shifting or non-sense mutations of the gene code for a truncated non-functional dystrophin protein without its cysteine domain [134]. The deficient dystrophin cannot act as a cohesive protein and results in the disaggregation of dystrophin-associated protein complex (DAPC), ultimately resulting in an impaired structure and reduced durability of the myofibres [133,135].

Most patients with DMD will ultimately develop dilated cardiomyopathy (DCM), one of the main causes of DMD-associated mortality [136]. Indeed, DMD-associated DCM is responsible for 2% of inherited DCM, often resulting in heart failure and ventricular arrhythmia [137]. It is characterised by inferolateral left ventricle regional contractile impairment and replacement fibrosis [138]. Its gradual progression with increased muscle stiffness and ventricular dilation ultimately results in more severe heart failure. The prognosis of the DMD patient with DCM is poor, with limited therapeutic options [139].

Several iPSC-based models have been established to recapitulate the pathophysiology of DMD-associated DCM, including calcium handling changes, impairment of myofibrils, and membrane fragility. After repeated stretch–contraction cycles, it was observed that the cell morphology was altered due to rupture of the sarcolemma. Compared with patient-derived tissues, iPSC models permit investigation of the early-stage pathogenesis of DMD and the different gene mutations associated with DMD-associated DCM, including Δ *Exon* 50, Δ *Exons* 45–52, *c.5899C>T*, and more [81,120,140].

The in vivo model can recapitulate the disease phenotypes and provide a more synoptic picture of the pathophysiology. The in vitro cellular models show the molecular changes observed in the tissues of the DMD patient, including the DMD signature dystrophin deficiency and the downstream changes, such as increased oxidative stress, impaired autophagy, and immunoproteasome dysregulation [118,140,141,142]. Studies show that the overall ATP production and basal respiration rate are decreased in the disease cell lines [117]. Furthermore, the yes-associated protein (YAP) is also modified due to altered actin stress fibers, leading to reduced proliferation of the iPSCs-derived cardiomyocytes. Cytosolic calcium has been found to be overloaded with increased intracellular Ca^2+^ concentration, especially after mechanical stretching [118]. Utilising high-throughput methods, muscle genes and mitochondrial metabolism genes have been identified to be dysregulated from the somite stage [119].

The molecular pathogenic profile can also be linked to the cardiac cardiomyopathic and electrophysiologic phenotype observed in affected patients. Patients with DMD-associated cardiomyopathy frequently suffer supraventricular and ventricular arrhythmias [143]. The diseased cell line has been shown to exhibit slower firing rates and increased events of delayed after depolarisations (DADs) and oscillatory prepotentials (OPPs). The length of the repolarisation also increased. This is especially the case in affected males, due to the increased *I*_Ca,L_ density. This explains the arrhythmic pattern observed in DMD patients [120].

After identifying the pathophysiological changes, novel drug targets can be identified to alleviate the disease phenotype. In a study with Δ *exons* 49–50, a proteasome inhibitor was applied to rescue the immunoproteasome dysregulation observed in iPSC-derived cardiomyocytes [7]. In another study of Δ *exons* 45–52, a membrane sealant Polaxamer 188 was able to restore the normal phenotype upon understanding how the damaged membrane could lead to dysregulated mechanosensitive channels [81,144]. Furthermore, the advancement of gene therapy may also enable the correction of the *DMD* phenotype. Human artificial chromosomes (HACs) have been shown to restore the dystrophin expression level and the normal phenotype [89].

### 6.2. Fabry Disease

Fabry disease is an X-linked multi-system lysosomal storage disorder with an incidence of around 0.04 to 0.25 per 10,000 males, and a higher incidence for the later-onset variant [145,146,147]. The disease mainly affects males, and heterozygous females have a spectrum of symptoms [148]. The causal mutation in Fabry disease is located at the *GLA* site and results in a defect or deficiency in the production of the alpha-galactosidase A enzyme (a-GAL A). a-GAL A is a lysosomal enzyme responsible for the removal of terminal alpha-galactosyl groups in glycolipids, particularly globotriaosylceramide (Gb3). Deficiency leads to the consequent accumulation of glycolipids in various tissues [149]. Progressive storage of these glycolipids leads to cellular dysfunction and irreversible damage, resulting in symptoms such as neuropathic pain, left ventricular hypertrophy, renal impairment, and angiokeratoma [150]. Some patients have the atypical late-onset cardiac variant of Fabry disease that presents as unexplained left ventricular hypertrophy without previous symptoms in middle-aged males [151]. Other cardiac manifestations include coronary insufficiency, valvular involvement, arrhythmia, and atrioventricular conduction problems [152]. There is no cure for Fabry disease, and the current standard of care is enzyme replacement therapy with intravenous administration of synthetic a-GAL A every other week [153].

**Figure 5:**
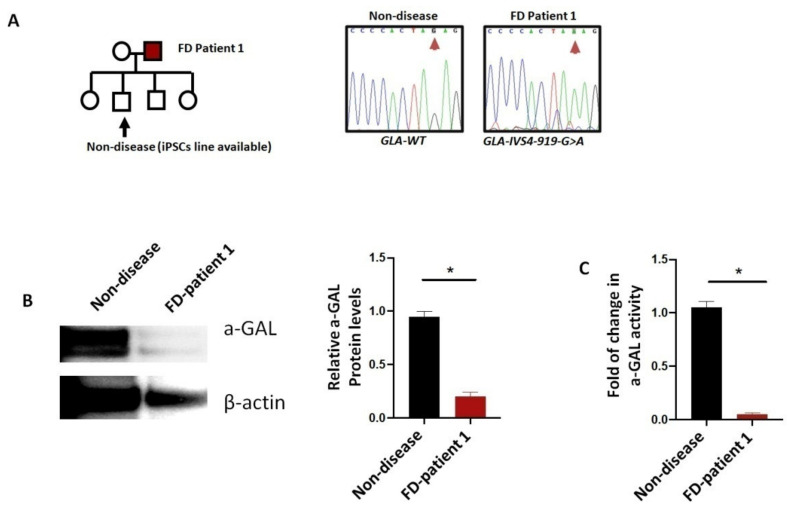
Generation of iPSC-derived cardiomyocytes from Fabry disease patient: (**A**) pedigree analysis of a family carrying the GLA-IVS4–919 G > A allele; (**B**) the iPSC line established from non-disease individual and FD-patient 1 were differentiated into cardiomyocytes and subjected to Western blot analysis; (**C**) a-GAL enzyme activity of the corresponding iPSC-derived cardiomyocytes. *: *p* < 0.05 between the indicated groups, N = 4.

Patient iPSC-derived cardiomyocytes can be used to model the disease and screen potential drugs [154]. Early attempts at modelling with iPSCs from patient fibroblasts generated a characteristic accumulation of Gb3 and pathological ultrastructural features [155]. Kuramoto Y. et al. [122] further isolated iPSCs from female patients and discovered individual clones that demonstrated either deficient or normal enzyme activity, that were then respectively used as the disease model and the isogenic control. Gb3 accumulation can be identified by mass spectrometry, and the expression of ANP in diseased iPSCs is increased, indicative of hypertrophy. Others have also generated iPSC-derived cardiomyocytes with decreased contractility, cellular hypertrophy, disturbed ion channel electrical currents, and peripheral displacement of cardiac contractile myofibrils, the typical phenotypic changes in diseased patients [121,128,156]. Currently, we are working with iPSCs established from a patient with Fabry disease. In brief, this patient contains a *GLA-IVS4–919-G>A* mutation with a significantly reduced a-GAL A protein level in the resultant cardiomyocytes (Figure 5).

Drug testing with patient iPSC-derived cardiomyocytes is made possible with the development of high-throughput compound screening [157]. No established data or drug testing research have been performed, but many studies have demonstrated the potential of the patient iPSC model for drug testing in Fabry disease. After identification of pathological phenotypes in mutant iPSCs, candidate drugs can be tested for normalisation or minimisation of these phenotypic changes and the drug toxicity can be assessed [158,159]. Several molecules, such as interleukin-18 and arachidonate 12/15-lipoxygenase, have been demonstrated in Fabry iPSC-derived cardiomyocytes to be associated with disease progression, and respective inhibitors have been proposed to have an adjunctive effect with enzyme replacement therapy [123,160]. Future investigations could make use of an iPSC-based model to test the effectiveness of these inhibitors in the alleviation of Fabry phenotypes.

### 6.3. Rett Syndrome

Rett syndrome is a monogenic X-linked dominant neurological disorder affecting 0.44 to 0.72 per 10,000 females aged 2 to 18 years, with similar rates across different races and ethnicities [126,161,162]. The disease mainly affects females, as mutations in hemizygous males often lead to embryonic or neonatal death in the first year of life [125]. Females with typical Rett syndrome initially develop normally after an uneventful full-term delivery but after several months experience loss of speech, purposeful hand movements, gait abnormalities, postnatal microcephaly, stereotypic hand movements, seizures, autistic features, and respiratory, cardiac, and nervous system abnormalities. Neurological development arrests by the age of one [156]. Some patients have atypical Rett syndrome that does not include all of the classic phenotypes seen in the typical form. Approximately 90% of the typical Rett syndrome and the milder preserved speech variant are due to a loss-of-function mutation in the *MECP2* gene, located in the long arm of the X-chromosome (Xq28) [163]. The *MECP2* gene encodes for methyl-CpG binding protein 2, which is abundantly expressed in the brain as an epigenetic modulator to control gene expression through DNA methylation [164]. A minority of patients with atypical Rett syndrome have mutations in the cyclin-dependent kinase-like 5 (*CDKL5*), Forkhead box protein G1 (*FOXG1*), myocyte-specific enhancer factor 2C (*MEF2C*), and transcription factor 4 (*TCF4*) genes [165].

Patients with Rett syndrome are at an increased risk of life-threatening cardiac arrhythmia with prolongation of QT corrected interval and alternations of ventricular repolarisation with the possibility of sudden unexpected cardiac death [166]. Other cardiac phenotypes include reduced cardiac vagal tone, sympatho-vagal imbalance, and heart rate variability [161].

Patient fibroblast-derived iPSCs have been used for disease modelling and drug testing in Rett syndrome. Rett-iPSCs from female patients retained the ability to undergo X-inactivation and differentiation into neurons. Compared with their wildtype controls, these diseased iPSC-generated neurons were smaller in size, and had fewer glutamatergic synapses, reduced spine density, smaller soma size, altered calcium signalling, and electrophysiological defects [128,162]. Diseased iPSC-derived neural cultures also resulted in changes to exosome protein cargo and signalling bioactivity [129]. The iPSCs generated from atypical Rett syndrome patients with a *CDKL5* mutation exhibited a decrease in axon outgrowth, dendritic morphogenesis, and synapse formation in diseased neurons [167].

A patient-derived iPSC model can also be used to screen the effects of candidate drugs by observation of the reversal of Rett disease phenotypes. IGF-1 and gentamicin administration have been found to be effective in the partial reversal of Rett-like symptoms in a cell culture, similar to results in a previously described mouse model [128]. Administration of control exosomes also rescues neurodevelopmental deficits seen in Rett syndrome iPSC models [129]. Selective inhibitors of histone deacetylase improved GABAergic circuit disruption and acetylated a-tubulin defects present in iPSC-derived neurons [130]. Traditional Indian *Medhya Rasayana* herbs have also been shown to reverse neurological disorders when tested in iPSC models [131].

### 6.4. Danon Disease

Danon disease is an X-linked dominant lethal multi-systemic condition that affects 1–4% of patients with hypertrophic cardiomyopathy [168]. It is caused by the mutation of the X-linked *LAMP2* gene that encodes lysosomal-associated membrane protein 2 (LAMP2). Deficiency in LAMP2 protein production leads to the failed fusion of autophagosomes and lysosomes, with the consequent accumulation of glycogen in muscle cells [169]. Male Danon disease patients manifest with myopathy, retinopathy, cognitive impairment, and hypertrophic cardiomyopathy (HCM) that ultimately results in sudden death in the absence of heart transplantation [170,171]. In heterozygous females, the age of onset of the disease is 15 years later than in males, and they mainly present with cardiac-restricted manifestations, including cardiomyopathy and arrhythmia [172].

To this end, several Danon disease patient-specific iPSC-based models have been established. Using this model, we have previously demonstrated that the accumulation of autophagy materials inside the affected cardiomyocytes is mainly due to the failure to clear the autophagic cargo, rather than over-activation of the autophagy signalling pathway [32].

In addition, increased mitochondrial oxidative stress, impaired mitophagy flux, and increased apoptosis have also been observed in iPSC-derived cardiomyocytes carrying the *LAMP2* mutant [173]. This results in the reduced OCR of basal respiration, ATP production, and maximal respiratory capacity (MRC) in the Danon disease iPSC-derived cardiomyocytes [174]. Treatment of the diseased iPSC-derived cardiomyocytes with antioxidant N-acetylcysteine has been shown to reduce the level of oxidative stress and alleviate the mitochondrial fragmentation pattern observed in the diseased cell line. Given the low rate of ATP generation, Danon disease iPSC-derived cardiomyocytes switched to utilising the glycolysis pathway to sustain their metabolism [175]. The Danon disease iPSC-derived cardiomyocytes exhibited downregulation of protein synthesis, potentially originating from reduced ATP synthesis and increased unfolded protein responses [175,176].

Danon disease iPSC-derived cardiomyocytes also exhibit diseased cardiac phenotypes, demonstrating an impaired contractile function similar to hypertrophic cardiomyopathy. It was shown that the contractility was decreased [32], and the contractile force per post was lower than that of control cell lines. The contractile force has been shown to return to normal after correction of the *LAMP2* mutation (c.247C>T) using the CRISPR-cas9system [174]. The LAMP2-deficient iPSC-derived cardiomyocytes exhibit a reduced amplitude of intracellular calcium transient and reduced maximum velocity of calcium release and reuptake. This is due to the reduced cellular level of calcium handling proteins in the mutant line, such as sodium–calcium exchanger (NCX1), sacro/endoplasmic reticulum Ca^2+^-ATPase 2, and ryanodine receptor 2 (RYR2) [32]. The Danon disease iPSC-CM also demonstrates traits of heart failure, such as significantly increased expression of ANP and BNP in the diseased cell line [127].

As the disease is due to phenotypic demonstration of the diseased allele, activating the previously silenced normal X-chromosome with the wildtype *LAMP2* gene will rescue the diseased phenotype in the heterozygous female Danon disease patient. To prove this concept, we have created isogenic iPSCs from female Danon disease patients. By reactivating the wildtype *LAMP2* allele by demethylation, we were able to restore LAMP2 protein production and improve the autophagic functions in the affected cells [32].

## 7. Isogenic iPSCs as a New Platform to Study X-Chromosome Inactivation

**Figure 6:**
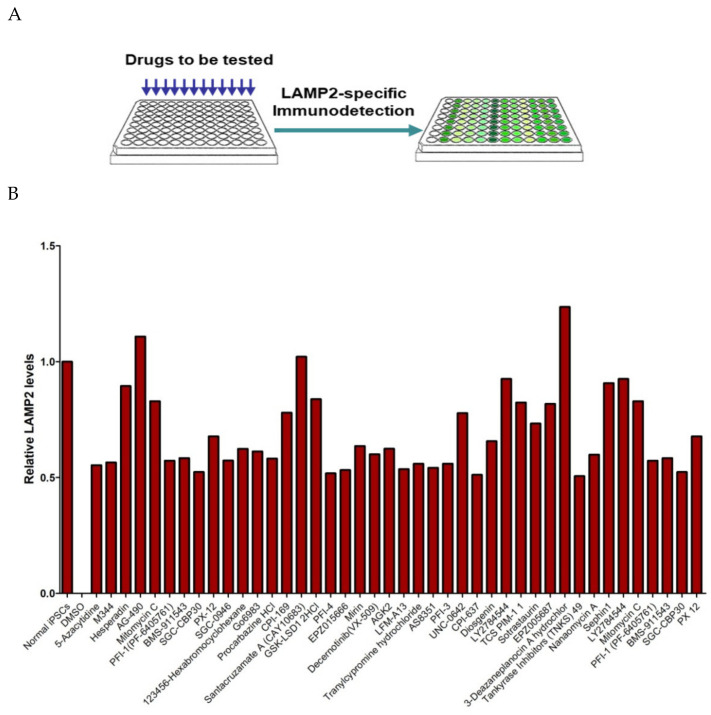
Application of the iPSCs with a silenced wildtype LAMP2 allele for drug screening: (**A**) the iPSCs with silenced LAMP2 allele were cultured on 96-well plates and treated with DiscoveryProbe™ Epigenetics Compound Library (APExBio). The reactivation of X-chromosomes will be detected using Alexa488-conjugated antibodies specific to human LAMP2; (**B**) primary screening results.

As mentioned before, isogenic iPSC-pairs with distinctive phenotypes can now be generated from a heterozygous female carrying one X-linked wildtype allele and one X-linked mutant allele. Making use of such a system, we have established an isogenic-iPSC-based platform to screen chemicals that can activate the silenced X-chromosome. In brief, as demonstrated previously [32], when the LAMP2-deficient iPSC carrying a wildtype *LAMP2* allele on the silenced X-chromosome was treated with a drug that could reactivate the silenced X-chromosome, cells started to produce a functional LAMP2 protein that could be detected by a simple immunoassay (Figure 6). Using this system, we have screened a library of approximately 300 chemicals and identified several inhibitors of the JAK2 signalling pathway (e.g., LY2784544 and BMS-911543) that can reactivate the genes on the silenced X-chromosome. This result not only facilitates the development of therapeutic targets for X-linked disorders, but also reveals the signalling pathways that may govern the randomised X-chromosome inactivation.

## 8. Limitations

Cardiomyocytes derived from patient-specific iPSCs offer an exclusive and convenient means to model disease phenotypes that are associated with X-linked mutations. Nonetheless, the functional immaturity of the iPSC-derived cardiomyocytes remains an important issue to be addressed. As demonstrated earlier by our group, compared with human ESC-derived cardiomyocytes, human iPSC-derived cardiomyocytes usually exhibit a reduced calcium handling ability and sarcoplasmic reticulum function [177]. This is due to the immature nature of the human iPSC-derived cardiomyocytes [178]. Human iPSC-derived cardiomyocytes display a lack of T-tubules and poor colocalisation of calcium channels and ryanodine receptors [179]. Therefore, extra attention should be paid when one considers modelling cardiomyopathies that resulted from the gene mutation that may affect calcium transients, such as DMD [180]. The immature iPSC-derived cardiomyocytes also demonstrate some different electrophysiology, cell morphology, and metabolism. As such, there is a need to improve the maturity of the iPSC-derived cardiomyocytes; this can be achieved with a prolonged culturing time [181], physical stimulation [182,183], biochemical stimulation [184], and metabolic alterations [185]. Moreover, since the iPSC-derived cardiomyocytes area contains a mixed population of atrial, ventricular, and nodal-like cells, these cells may differ substantially in their electrophysiological properties despite being genetically identical. Thus, for modelling cardiomyopathies associated with ion channel abnormalities, one may need to consider the contribution of the different cardiomyocyte subtypes to the results obtained or derive sub-population specific cardiomyocytes in the differentiation process. Similarly, when testing the effects of a putative antiarrhythmic drug, the immature phenotype and mixed cardiomyocyte subtypes should also be taken into account. Finally, although the use of isogenic iPSCs may help to evaluate the effects of a particular mutation under the same genetic background, the results may still not be sufficient to help predict prognosis and disease severity in heterozygous female patients.

## Figures and Tables

**Table 1:** X-linked genes that cause cardiomyopathy.

Disease	Gene	Type of Cardiomyopathy	Extra-Cardiac Manifestations
Duchenne muscular dystrophy	*DMD*	Dilated [10]	Muscle weakness [11] Growth delay [12]
Fabry disease	*GLA*	Hypertrophic [13]	Neuropathic pain Renal impairment Angiokeratoma [14]
Familial cardiac filaminopathy	*FLNA*	Dilated [15]	Periventricular heterotopia [16]
		Hypertrophic [17]	
		Both dilated and hypertrophic [18]	
Danon disease	*LAMP2*	Hypertrophic [19]	Skeletal myopathy Retinopathy [20] Cognitive impairment [21]
		Dilated [22] Left-ventricular non-compaction [23]	
Rett syndrome	*MECP2*	Arrhythmogenic [24]	Extrapyramidal motor dysfunction [25] Epilepsy [26] Bone fracture [27]
X-linked myotubular myopathy	*MTM1*	Dilated [28]	Respiratory failure [29] Muscle weakness

**Table 2:** Application of iPSCs in the investigation of X-linked cardiomyopathies.

Disease	Pathophysiological Changes	References
Duchenne muscular dystrophy	Express truncated non-functional dystrophin protein, disrupted myofibrils, calcium overloads, disrupted membrane fragility, increased DAD and OPPs	[7,117,118,119,120]
Fabry disease	Gb3 accumulation, deficient enzyme, high ANP expression Decreased contractility, cellular hypertrophy, disturbed ion channel electrical currents, peripheral displacement of myofibrils	[121,122,123]
Danon disease	Deficiency of LAMP2, accumulation of autophagy materials, increased mitochondrial oxidative stress, increased apoptosis, altered metabolism, impaired contractile function, reduced calcium transients	[32,124,125]
Rett syndrome	Decreased soma size, fewer glutamatergic synapses, reduced spine density, altered calcium signalling and electrophysiological defects, decreased axon outgrowth, dendritic morphogenesis Changes in exosome protein cargo and signalling bioactivity	[124,125]

**Table 3:** Applications of iPSC-based models for drug testing.

Disease	Drug/Treatment Tested	References
Duchenne muscluar dystrophy	Proteasome inhibitors, polaxamer188, human artificial chromosomes	[7,81,89]
Fabry disease	glucosylceramide synthase inhibitor	[126]
Danon disease	N-acetylcystein, rotenone, DNA-demethylating drugs	[32,127]
Rett syndrome	IGF-1, gentamycin, exosomes hstone deacetylase inhibitors, medhya rasayana	[128,129,130,131]

## Data Availability

Not applicable.
